# Influence of helium-ion bombardment on the optical properties of ZnO nanorods/p-GaN light-emitting diodes

**DOI:** 10.1186/1556-276X-6-628

**Published:** 2011-12-12

**Authors:** Naveed ul Hassan Alvi, Sajjad Hussain, Jen Jensen, Omer Nur, Magnus Willander

**Affiliations:** 1 Department of Science and Technology (ITN), Campus Norrköping, Linköping University, 60174 Norrköping, Sweden; 2 Department of Physics, Chemistry and Biology, Linköping University, 58183, Linköping, Sweden

## Abstract

Light-emitting diodes (LEDs) based on zinc oxide (ZnO) nanorods grown by vapor-liquid-solid catalytic growth method were irradiated with 2-MeV helium (He^+^) ions. The fabricated LEDs were irradiated with fluencies of approximately 2 × 10^13 ^ions/cm^2 ^and approximately 4 × 10^13 ^ions/cm^2^. Scanning electron microscopy images showed that the morphology of the irradiated samples is not changed. The as-grown and He^+^-irradiated LEDs showed rectifying behavior with the same I-V characteristics. Photoluminescence (PL) measurements showed that there is a blue shift of approximately 0.0347 and 0.082 eV in the near-band emission (free exciton) and green emission of the irradiated ZnO nanorods, respectively. It was also observed that the PL intensity of the near-band emission was decreased after irradiation of the samples. The electroluminescence (EL) measurements of the fabricated LEDs showed that there is a blue shift of 0.125 eV in the broad green emission after irradiation and the EL intensity of violet emission approximately centered at 398 nm nearly disappeared after irradiations. The color-rendering properties show a small decrease in the color-rendering indices of 3% after 2 MeV He^+ ^ions irradiation.

## Introduction

Zinc oxide (ZnO) with bad gap of 3.37 eV has very attractive properties to play a major role in nanoscale electronic and optoelectronic devices. Its excellent properties combined with the easiness of growing it in the nanostructure form have made it one of the most attractive and versatile semiconductor [1,2]. It has both semiconducting and piezoelectric properties and in addition it is biocompatible and bio-safe. ZnO possesses deep level emission (DLE) bands emitting all the colors in the visible region and has good color-rendering properties [3-5].

Among all of the known oxide semiconductors, ZnO nanorods (NRs) are the best choice for intrinsic white light emission due to their easy growth via chemical as well as other physical vapor-phase approaches [6]. ZnO NRs with small footprint and large surface area to volume ratio are good candidates for heterojunction light-emitting diodes (LEDs) as compared to thin films. This is due to the fact that the stress/strain due to lattice mismatch can easily be released for nanorods when compared to thin films. In addition, a general property of NRs-based LEDs is that each nanorod can act as a wave guide, minimizing side scattering of light, thus enhancing light emission and extraction efficiency [7]. It is still a challenge to achieve a reproducible, high quality p-type epitaxial technology for ZnO. This hinders the progress of ZnO homojunction LEDs. The alternative way is to grow n-type ZnO nanostructures on top of other p-type substrates to make heterojunctions [8-10]. The close lattice match is the main factor that can influence the optical and electrical properties of heterojunctions. The p-GaN as a substrate is a good candidate that has small lattice mismatch with ZnO. There is only few growth reports of n-ZnO nanorods on p-GaN, and on white LEDs based on them available in the literature, e.g., [11-13].

The properties of a material can be changes by irradiation of that material with energetic particles such as electrons or ions that normally gives rise to formation of defects in the target material [14]. An important consideration for space and nuclear applications of ZnO nanostructures based LEDs is that these LEDs should be reliable to withstand and to operate in radiation hard conditions. In this paper, we have investigated the effect of 2 MeV He+ ion irradiation on the optical properties of ZnO nanorod-based LEDs. There are only few reports about the effect of high-energy electrons irradiation that has been reported for ZnO [15], GaN [16,17], and SiC [18]. Effect of ion and electron irradiation on the properties of nanostructured materials has been studied [14]. Much less data are available regarding the effect of heavier particles (such as He+ ions) on the physical properties of ZnO nanostructures [19]. The irradiation changes the amount of defects in ZnO nanorods resulting in changes in the optical properties of ZnO. The effect of the ion irradiation of complete device, like a LED, has not been studied so much.

## Experimental details

ZnO NRs were grown on p-GaN substrates by the vapor-liquid-solid (VLS) mechanism. Gold nano particles are used as catalyst for the growth. A thin film of gold with thickness of approximately 4 nm was deposited on the substrates in a low-vacuum metallization chamber. In this method, pure zinc powder (99.9%) is used as the source material. The pure zinc powder was placed in a quartz tube and the substrates are placed on the boat at the downstream side of the gas flow. The substrates are placed at a distance of 1 to 2 cm away from the zinc powder. The mixture of the argon and oxygen gases with a ratio of 8:1 is introduced in the quartz tube. Argon was applied as a carrying gas and oxygen was as reactant gas [20]. The growth temperature was approximately 680°C.

After the growth of the ZnO NRs on p-GaN substrates, three samples were used to process the LEDs. Pt/Ni/Au alloy was used to form ohmic contact to the p-GaN substrate. The thickness of the Pt, the Ni, and the Au layers were 20, 30, and 80 nm, respectively. The sample was annealed at 350°C for 1 min in flowing argon gas atmosphere. This alloy gives a minimum specific contact resistance of 5.1 × 10^-4 ^Ω cm^-2 ^[21]. After that an insulating photo-resist layer was spun coated on the ZnO NRs to fill the gaps between the NRs to isolate electrical contacts on the ZnO NRs from reaching the p-type substrate and helps to prevent the carrier cross talk among the nanorods. To form the top contacts, the tips of the ZnO NRs were exposed by using plasma ion etching after the deposition of the insulating photo-resist. Non-alloyed Pt/Al metal system was used to form the ohmic contacts to the ZnO NRs. The thickness of Pt and Al layers were 50 and 60 nm, respectively. This contact gives a minimum specific contact resistance of 1.2 × 10^-5 ^Ω cm^-2 ^[22]. The diameter of the top contact was about 0.58 mm. Two of these fabricated LEDs and two GaN substrates with the as-grown ZnO NRs were are irradiated by using 2.0 MeV He^+ ^ions with fluences of approximately 2 × 10^13 ^ions cm^-2 ^and approximately 4 × 10^13 ^ions cm^-2^. All the samples were irradiated at room temperature under normal incidence at the Tandem Laboratory, Uppsala University Sweden. The ion beam flux was at about 6 × 10^10 ^ions s^-1 ^cm^-2 ^and the beam spot was roughly 1 cm^2^. The projected ion range is calculated by the SRIM2008 code [23] to be 4.9 μm assuming a density for ZnO of 5.6 g/cm^3^, so the major part of the whole ZnO nanorods were influence by the beam. The irradiated ZnO nanorods are used for PL measurements and the devices were used to measure the IV and the electroluminescence (EL) measurements. The PL measurements were performed at room temperature. Laser lines with a wavelength of 266 nm from a diode laser pumped resonant frequency doubling unit (MBD266) were used as an excitation source. The EL measurements of the fabricated LEDs were performed by using a photomultiplier detector at room temperature. The spectra were measured from the top contacts of the LEDs by detecting the light escaping from the edge of the top contact electrode.

## Results and discussions

**Figure 1:**
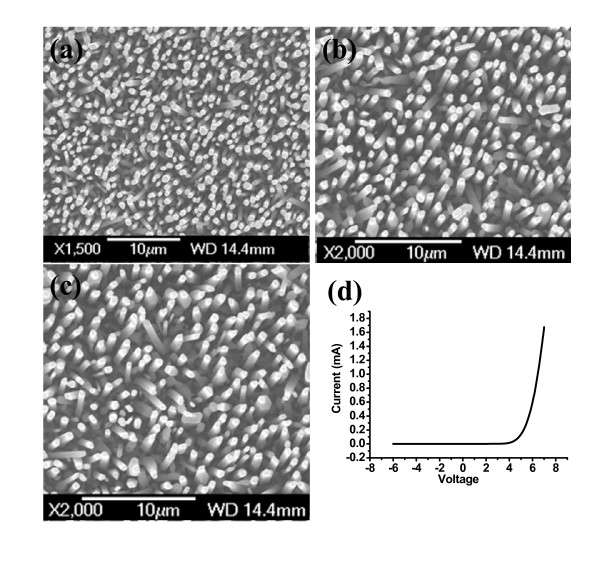
**SEM image of ZnO nanorods on p-GaN substrate**. (**a**) As grown, (**b**) after irradiation with fluency of approximately 2 × 10^13 ^ions/cm^2^, (**c**) after irradiation with fluency of approximately 4 × 10^13 ^ions/cm^2^, and (**d**) typical I-V characteristics for the fabricated LEDs.

The morphology and size distribution of the as-grown ZnO nanorods were investigated by using JEOLJSM-6301F SEM. The top SEM view of as-grown and irradiated ZnO nanorods are shown in Figure 1a, b, c, respectively. The ZnO structures had a uniaxial orientation of <0001> perpendicular to the substrates. The epitaxial growth of the ZnO nanorods with respect to the p-GaN substrates, forming n-ZnO-(nanostructures)/p-GaN p-n heterojunction. From the SEM images, the mean diameters of our as-grown ZnO nanorods was approximately 450 nm. The approximate length of ZnO nanorods was 3 μm.

The I-V characterization of the fabricated LED is shown in Figure 1d. The irradiated and non irradiated LEDs have same I-V curves. The I-V curve shows rectifying behavior as expected from these LEDs. It indicates clearly that reasonable p-n heterojunctions characteristics were achieved. The turn-on voltage of these heterojunctions LEDs is around 3 V.

**Figure 2:**
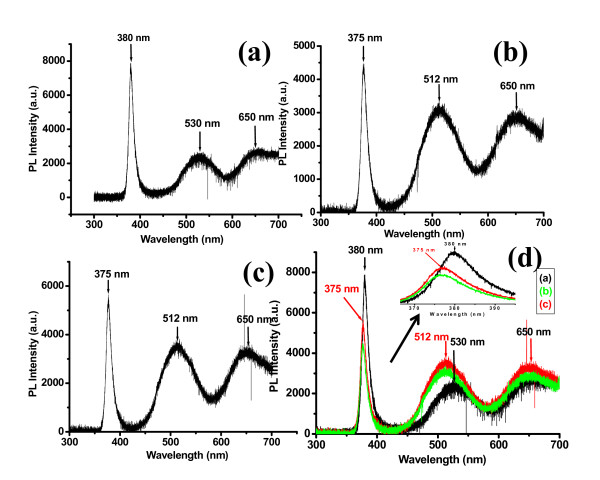
**Room-temperature photoluminescence spectrum for ZnO nanorods**. (**a**) As grown, (**b**) after irradiation with fluency of approximately 2 × 10^13 ^ions/cm^2^, (**c**) after irradiation with fluency of approximately 4 × 10^13 ^ions/cm^2^, and (**d**) the PL spectra of all the samples together for comparison.

**Figure 3:**
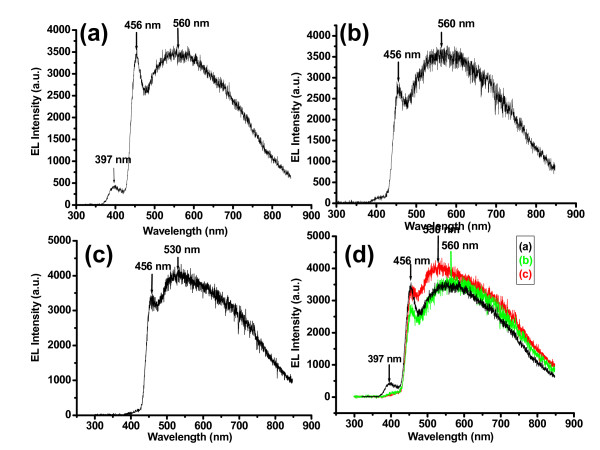
**Displays the electroluminescence spectra for n-ZnO nanorods/p-GaN LEDs, (a) As grown, (b) after irradiation with fluency of approximately 2 × 10^13 ^ions/cm^2^, (c) after irradiation with fluency of approximately 4 × 10^13 ^ions/cm^2^, and (d) the EL spectra of all the LEDs together for comparison**.

Figure 2a, b, c shows the photoluminescence of the as-grown and irradiated ZnO NRs. Figure 3a shows the PL spectrum for as-grown ZnO NRs. The band-edge emission and the DLE peaks are observed at approximately 380 nm (3.26 eV) and 530 nm (2.33 eV). The observation of the band-edge emissions at 380 nm (3.26 eV) are attributed to the first and second longitudinal optical (LO) phonon replica. This is consistent with the LO-phonon energy of 72 meV for ZnO. Their accurance shows that our as-grown ZnO structures are of good crystalline quality [24]. The green emission centered at 2.33 eV (530 nm) is attributed to recombination between the bottom of the conduction band to the O_i _energy level and it is approximately agreed with reported data for the transition energy from bottom of the conduction band to O_i _energy level (approximately 2.28 eV) [25,26].

Figure 2b, c shows the PL spectrum for irradiated ZnO nanorods with fluencies of approximately 2 × 10^13 ^ions/cm^2 ^and approximately 4 × 10^13 ^ions/cm^2^, respectively. The band-edge emission and the DLE peaks are observed at approximately 375 nm (3.30 eV), and 512 nm (2.42 eV). The green peak centered at 2.42 eV (512 nm) is attributed to recombination between the conduction band energy level to the V_O _energy level and it is approximately consistent with the transition energy from Zn_i _energy level to V_O _energy level (approximately at 2.47 eV). A comparison of the PL spectra of the ZnO NRs before and after the He^+ ^ion irradiation is shown in Figure 2d. It shows that the PL intensity of the near-band-edge decrease and the PL intensity of the deep level emission increase after the He^+ ^ion irradiation. The decrease in the UV emission is due to degradation in the crystalline quality after the He^+ ^ion irradiation. The enhancement in the green emission is due to the increase of radiative defects such as oxygen vacancies [27].

It is also observed that there is a blue shift of about 0.0347 eV in the near-band-edge emission after the He^+ ^ion irradiation. The blue shift in the excitonic emission peak can be attributed to the presence of homogeneous compressive strain produced by the He^+ ^ion irradiation. Compressive strain increases the band gap and affects the optical properties of the materials [28]. There is also a blue shift of 0.082 eV in the green emission after He^+ ^irradiation. This blue shift is due to the fact that the He^+ ^ion irradiation increases the oxygen vacancy defects as compared to oxygen interstitials defects in the ZnO. The green emission is the superposition of the emissions due to oxygen vacancies and oxygen interstitials. The oxygen interstitials have lower energy (2.28 eV) as compared to oxygen vacancies (2.47 eV). The as-grown ZnO nanorods have green emission approximately centered at 2.33 eV and it is very close to energy of oxygen vacancies but after He^+ ^irradiation oxygen vacancies defects increase and irradiated ZnO nanorods have green emission is centered at approximately 2.42 eV and it is very close to energy of oxygen vacancies.

The orange-red emission peak is centered at 650 nm (1.90 eV). This orange-red emission can be attributed to the transition from zinc interstitial (Zn_i_) to oxygen interstitial (O_i_) defect levels in ZnO [26]. By using the full potential linear muffin-tin orbital method, which explains that the position of the O_i _level is located approximately at 2.28 eV below the conduction band and the Zn_i _level is theoretically located at 0.22 eV below the conduction band. Therefore the transition energy from Zn_i _to O_i _levels is approximately 2.06 eV [26]. This value agrees approximately with the experimental (EL) peak centered at 1.90 eV.

Figure 3a shows the EL spectra of as-grown ZnO nanorods. The violet, violet-blue, and green emissions are observed and are centered approximately at 397 nm (3.12 eV), 456 nm (2.71 eV) and 560 nm (2.21 eV), respectively. It was reported that the violet emission from undoped ZnO nanorods corresponds to zinc interstitials (Zn_i_) [26]. The violet peak is centered at 3.12 eV (397 nm) and it agrees well with the transition energy from the valence band to the Zn_i _level in ZnO (approximately 3.1 eV). The violet-blue peak centered at 2.71 eV (456 nm) is attributed to recombination between the Zn_i _energy level to the V_Zn _energy level and it is approximately agreed with the transition energy from Zn_i _energy level to V_Zn _energy level (approximately 2.84 eV). There is a difference of 0.13 eV. Maybe this difference is due to the effect of GaN substrate as GaN also emits blue light. As the violet-blue peak is not observed in the PL spectra this may support the argument that the blue emission is from GaN substrate. The green emission centered at 560 nm (2.21 eV) is attributed to oxygen interstitials and is discussed above. The green emission in the EL spectra is red shifted as compared to the PL spectra. It may be due to the heating effect of the device.

Figure 3b, c shows the EL spectra of He^+ ^ion irradiated LEDs. The EL spectra of irradiated LEDs shows that the violet peak centered at 397 nm (3.12 eV) almost disappeared after He^+ ^ion irradiation. It disappears due to the poor crystalline quality after irradiation. The green emission EL peak is blue shifted about 0.125 eV. The reason of this blue shift in the green peak is discussed above. The blue emission looks stable as it is from the substrate.

**Figure 4:**
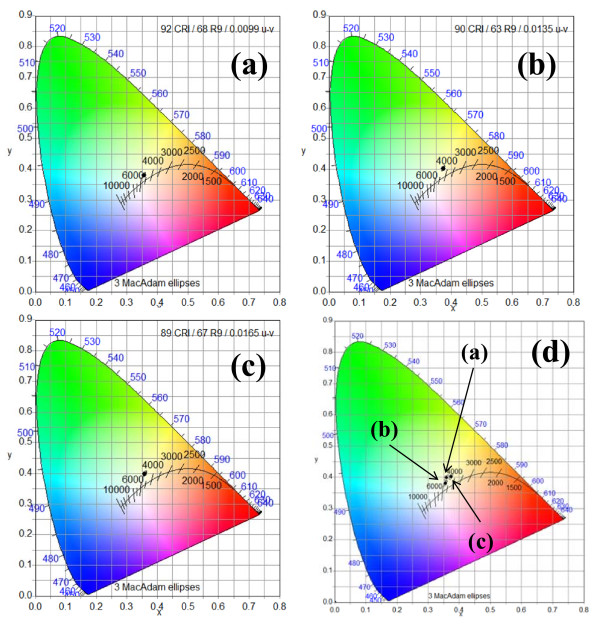
**Shows the CIE 1931 *x*, *y *chromaticity space**. Showing the chromaticity coordinates of LEDs under forward bias for ZnO NRs/p-GaN LEDs, (**a**) as-grown ZnO NRs, (**b**) after irradiation with fluency of approximately 2 × 10^13 ^ions/cm^2^, (**c**) after irradiation with fluency of approximately 4 × 10^13 ^ions/cm^2^, and (**d**) all together for comparison.

Figure 4a, b, c shows the CIE 1931 color space chromaticity diagram in the (*x*, *y*) coordinates system. The chromaticity coordinates of the non radiated and He^+ ^ion radiated with fluencies of approximately 2 × 10^13 ^ions/cm^2 ^and approximately 4 × 10^13 ^ions/cm^2 ^LEDs are (0.3557, 0.3805), (0.3735, 4020), and (03594, 0.3983) with correlated color temperatures (CCTs) of 4, 745, 4, 345, and 4, 708 K, respectively. It seems that the chromaticity coordinates for non radiated LED are close to Planckian locus (about 2 Mac-Adam ellipse away) and can be called white light according to the US standard ANSI_ANSLG C78, 377-2008 for the solid-state light sources which determines that the distance of 3 Mac-Adam ellipses form Planckian locus can be allowed for white light. The He+ ion-radiated LEDs are slightly decreased from the Planckian Locus and are very close to white light. They are about 4 Mac-Adam ellipses away from the Planckian Locus. The color-rendering indices are 92, 90, and 89 for the non-radiated and He^+ ^ion radiated with fluencies of approximately 2 × 10^13 ^ions/cm^2 ^and approximately 4 × 10^13 ^ions/cm^2 ^LEDs, respectively. It shows that there is only a small effect (3%) on the optical properties. So this might be a good observation for space and nuclear applications. The 2-MeV ions only have a minor effect on optical properties of the LED devise after irradiation with moderate fluences.

## Conclusion

In summary, the influence of He^+ ^ion irradiation on the optical properties of ZnO nanorods has been investigated as a possible candidate for space applications of ZnO nanorods based LEDs. The PL investigations show that the irradiation has influence the high-energy defects in the ZnO, especially the defects responsible for UV, violet and green emission in ZnO. Due to this influence crystallinity in ZnO decreases and as a result PL intensity of ultraviolet (UV) emission decreases. A blue shift in UV and green emission was found. The EL spectra reveal the same blue shift in the green emission as observed from the PL spectra. The irradiation has a minor effect on the color-rendering properties of the LEDs. It decreases the color-rendering indices from 92 to 89.

## Competing interests

The authors declare that they have no competing interests.

## Authors' contributions

All authors contributed equally. All authors read and approved the final manuscript
